# Spatial distribution of trace elements and ecotoxicity of bottom sediments in Rybnik reservoir, Silesian-Poland

**DOI:** 10.1007/s11356-016-6678-1

**Published:** 2016-05-25

**Authors:** Agnieszka Baran, Marek Tarnawski, Tomasz Koniarz

**Affiliations:** 1 Department of Agricultural and Environmental Chemistry, University of Agriculture in Krakow, Al. Mickiewicza 21, Krakow, 31-120 Poland; 2 Department of Hydraulic Engineering and Geotechnics, University of Agriculture in Krakow, Al. Mickiewicza 24/28, Krakow, 30-059 Poland

**Keywords:** Bottom sediment, Spatial distribution, Trace elements, Biotest, Ecological risk assessment

## Abstract

The aim of study was to integrate chemical analyses and toxicity bioassays in order to assess the environmental risk connected with the presence of trace elements in the sediments. This study examined the ecological significance of trace elements in bottom sediments by applying a set of complementary sediment quality assessment methods sediment quality guidelines (SQGs) (mean probable effect concentration quotient (PECQ)), potential ecological risk index (PERI), contamination degree (*C*_d_) and two bioassays: the bacterial luminescence inhibition test with *Vibrio fischeri* on sediment elutriates and the direct contact test with the ostracod crustacean *Heterocypris incongruens*. The samples were collected from 50 stations of Rybnik reservoir. The reservoir is a region with enormous concentration of industry, mainly hard coal mining, electric power industry, and transportation. Despite the high diversity in metal concentration in the sediments, the spatial distribution of trace elements in the sediments was very similar. Moreover, the strong positive correlations between individual pairs of trace elements indicate that they may derive from a similar source and move together. According to mean PECQs, 68 % of the samples were potentially non-toxic and 32 % of the samples were potentially toxic. PERI values suggested that 70 % of the sediment sampling sites exhibited low ecological risk from metal pollution while 24 % of the samples had severe and serious risk. Based on our combined evaluation, we believe that Cd and Cu in the sediment samples frequently caused adverse biological effects. Higher toxic responses were observed in the Microtox test than in the Ostracodtoxkit test. All the sediment samples were found toxic to *V. fischeri*, and 96 % of the samples had effect percentages >50 %. For *H. incongruens*, 12 % of the sediments were not toxic and 44 % had effect percentages >50 %. In order to perform a complex assessment of the environmental impact of metal pollution, both chemical and ecotoxicological analysis should be carried out.

## Introduction

Bottom sediments are an important part of the aquatic environment; they have numerous functions: ecological, geochemical, and economic. On the other hand, they are the final deposition place of various pollution (Förstner and Salomons 2010, Farkas et al. 2007; Du Laing et al. 2009; Fang et al. 2012; Rinklebe and Shaheen 2014). Pollution with trace elements is regarded as a serious threat to the environment and wildlife habitats due to their toxicity, persistence, and ability to be incorporated into food chains (Ayas et al. 2007; Gao et al. 2013; Urbaniak et al. 2008). Nowadays, key studies on trace elements in bottom sediments cover potential ecological risk, geochemical cycling, assessment of health risk caused by trace elements, and toxicity assessment (Fang et al. 2012; Bastami et al. 2015; Sayed et al. 2015; Li 2014, Baran and Tarnawski 2015). Different environmental factors such as chemical, physicochemical, biological, and ecotoxicological must be taken into account for the assessment of ecological risk caused by trace element exposure in the aquatic ecosystem. Moreover, all these factors should be integrated. Different methods have been applied in order to quantitatively assess the cumulative ecological risks associated with metals: geoaccumulation index (*I*_geo_), sediment enrichment factor (SEF), potential ecological risk index (PERI), excessive regression analysis, sediment quality guidelines (SQGs), Pollution Load Index (PLI), Risk Assessment Code (RAC), etc. (Burton 2002; Farkas et al. 2007; Guo et al. 2010; Ingersoll et al. 2001; Suresh et al. 2012; Fang et al. 2012; Wang et al. 2012; Hou et al. 2013; Fiori et al. 2013; Veses et al. 2013; Fu et al. 2013; Li 2014; Baran and Tarnawski 2015; Shaari et al. 2015; Sayed et al. 2015). A number of studies have indicated that a battery of bioassays is a good tool to assess ecological risk (Davoren et al. 2005; Mankiewicz-Boczek et al. 2008; Narracci et al. 2009; Tuikka et al. 2011; Gonçalves et al. 2013; Kemble et al. 2012; Besser et al. 2014; Baran and Tarnawski 2013, 2015). At the same time, it needs to be emphasized that the issue of the quality of bottom sediments and their significance in the evaluation of the condition of the aquatic environment is becoming more and more important. This is because the direct relation between bottom sediment quality and the ecological potential and the state of water pollution is indisputable. Many researchers have found that sediments are an indicator of water pollution and distribution of trace elements in sediments, and can reflect the water pollution level (Guo et al. 2010; Gonçalves et al. 2013; Sayed et al. 2015).

The aims of this study were as follows: (i) to investigate the concentration and spatial distribution of trace elements (Zn, Cd, Pb, Cu, Ni, Cr, Fe, Mn) in sediments, (ii) to evaluate the ecotoxicity of the sediments, and (iii) to integrate chemical analyses and toxicity bioassays in order to assess the environmental risk connected with the presence of trace elements in the sediments.

## Material and methods

### Study area

**Fig. 1:**
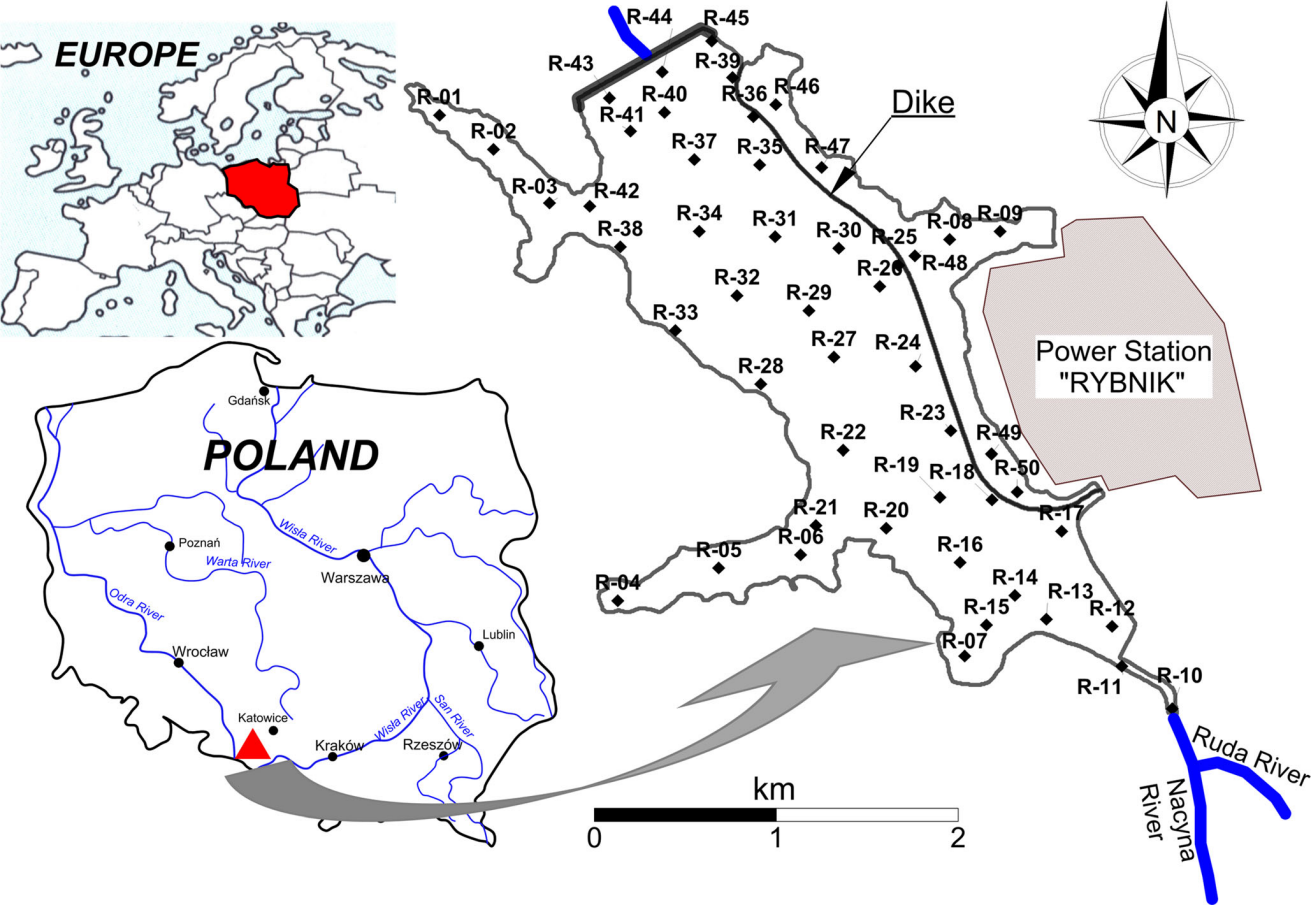
Localization of the reservoir and 50 station samples

The Silesian area (the southern part of Poland) is one of the key urban centers in the country, integrating 14 largest cities in the Silesia and Zaglębie regions and two million inhabitants over a surface area of 1200 km^2^ (Fig. 1). Moreover, the Silesian area is a region with enormous concentration of industry, mainly hard coal mining, electric power industry, transportation, coking and briquetting plants, and companies producing machinery, metals, chemicals, and building materials. The Rybnik reservoir is located in the center of the Rybnik Coal Region, one of the main industrial centers of Poland (Loska and Wiechuła 2003, Wiechuła et al. 2005). The concentration of industry in this area places Rybnik as significant to the Polish city in respect of the emission of gaseous and industrial solid waste produced. For this reason, strong attention is directed to the research of Upper Silesian area surface water contamination.

The Rybnik reservoir was created by building a frontal dam in 1972, partitioning the Ruda river at 23 + 584 km of its course. This structure is located at a height of 220.00 m above sea level in Rybnik-Stodoła. The reservoir dam closes the catchment area of the reservoir, which covers 308 km^2^. The reservoir is 7 km long. The reservoir initial capacity was 25.8 Mm^3^ at the impoundment area together with lateral impoundments of 555 ha. Rybnik reservoir is characterized by low water flow; each year the water changes about three to four times (Wiechuła et al. 2005). The main function of the reservoir is used water for surface cooling of water discharged from the condensers of Rybnik Power Station S.A. and flood control. Water temperature significantly affects a number of processes occurring in the reservoir, including the increase in mobility of trace elements (Wiechuła et al. 2005). The reservoir also provides benefits as a site for recreation and as a waterfowl habitat (Baran et al. 2015). Results of research carried out by the Institute of Meteorology and Water Management—National Research Institute have shown that the Rybnik reservoir is silting up very slowly. After 27 years of its operation, the level of silting reached 0.74 %, and after 36 years, it increased only to 0.97 %. The state of bottom sediment pollution poses a serious hazard to the environment due to industrial character of the Ruda river catchment. The sediments contain elevated content of heavy metals, and the most heavily polluted is the 150-ha zone in the north-western part of the reservoir. Maximum metal concentrations determined in this area were as follows: 902 and 1077 mg Zn kg^−1^ d.m. (dry mass), between 110 and 681 mg Cu kg^−1^ d.m., between 110 and 688 mg Cd kg^−1^ d.m, between 73.7 and 125 mg Pb kg^−1^ d.m, between 5.0 and 16.8 mg Cd kg^−1^ d.m., between 28.6 and 47.3 mg Ni kg^−1^ d.m, and between 125 and 197 mg Cr kg^−1^ d.m. (Baran and Tarnawski 2015).

### Sediment sampling

The samples of bottom sediments form Rybnik reservoir were collected from 50 stations (Fig. 1). The top layer of the sediment was collected from a depth of 0–15 cm using an Ekman sampler. After the decantation of overlying water, all sediment samples were refrigerated until analyzed.

### Chemical analyses

The sediment samples were analyzed for parameters such as total trace element concentration, grain size fractions, pH_KCl_, content of total carbon, and total nitrogen. Total element concentration (Zn, Cd, Pb, Cu, Ni, Cr, Fe, Mn) in the sediments was assessed after hot digestion in a mixture of HNO_3_ and HClO_3_ (3:2 *v*/*v*) acids (suprapure, Merck). Metal concentrations were analyzed using inductively coupled plasma atomic emission spectroscopy (ICP-AES) method on Optima 7300 DV (Perkin-Elmer) (Baran and Tarnawski 2013; Koniarz et al. 2014). The total Hg analysis was carried out using the atomic absorption spectrophotometer (AAS) for mercury determination (Advanced Mercury Analyser; AMA 254). The grain size fractions were determined according to the aerometric method. The total organic carbon (TOC) and total nitrogen (TN) content in the sediments were determined using an Elementar Vario MAX cube CNS analyzer. pH was measured at a 1:2.5 sediment to liquid ratio with 1 mol KCl dm^−3^. Sediment samples were analyzed in triplicates for which the relative standard deviations (%RSDs) were less than 10 % for the trace elements. Accuracy of the performed analyses was tested using reference material CRM 16-05 (trace elements) and Soil Standard Loamy (OAS) batch no. 133505 (organic carbon and total nitrogen). The results showed that the percentage of recovery ranged from 95 to 112 % for Zn, from 81 to 98 % for Cu, from 98 to 114 % for Pb, from 88 to 99 % for Cr, from 78 to 98 % for Cd, from 96 to 107 % for Ni, from 88 to 105 % for Hg, from 103 to 107 % for Mn, from 85 to 102 % for Fe, from 91 to 101 % for N, and from 101 to 105 % for C.

### Ecotoxicity tests

Toxicity assessment of bottom sediments was conducted using the Microtox® and Ostracodtoxkit F. Microtox® uses the bioluminescent properties of *Vibrio fischeri*. In this test, a decrease in the luminescence of the bacterium in an experimental sample is compared to that of the control (bacteria in a 2 % solution of NaCl). Elutriates from the sediment were prepared by mixing one volume of the sediment with four volumes of redistilled water and shaking mechanically for 24 h (Baran and Tarnawski 2013). After that time, the samples were centrifuged for 10 min at a speed of 3000 rpm and filtered. A standard test procedure was applied for sediment elutriate: 81.9 % screening test. Luminescence was measured before and after a 15-min incubation of the bacterial suspension with the studied sample. The analysis of measurement of the change in luminescence was performed on a Microtox M500 Analyzer (MicrobicsCorporation 1992). Toxicity determination of bulk samples was performed using the Ostracodtoxkit F. This test was used to measure the mortality and growth inhibition of the crustacean *Heterocypris incongruens* hatched from cysts after six days of exposure to the sediment samples. After six days of contact with the sediment, the percentage mortality and growth of the crustaceans were determined and compared with the results obtained in the (non-toxic) reference sediment (Ostracodtoxkit 2001; ISO 14371:2012).

The tests were conducted in accordance with the procedure recommended by the manufacturer (MicrobicsCorporation 1992; Ostracodtoxkit 2001; ISO 14371:2012). Four replicate samples were tested. In the Ostracodtoxkit, mortality percentage in the control at the end of the test was 0 %; thus, the validity criterion of “less than 20 % mortality in the control” was achieved. According to the operational procedure, the length increase of *H. incongruens* in the controls had to be at least 400 μm at the end of the test (Ostracodtoxkit 2001). The mean final lengths in the control range from 650 to 750 μm. When evaluating the test results, the following toxicity criteria were adopted (Persoone et al. 2003): non-toxic samples, percentage effect (PE) <20 %; slightly toxic samples 20 % ≤ PE < 50 %; toxic samples 50 % ≤ PE < 100 %; and highly toxic samples, PE = 100 %.

### Data analysis

#### Indexes of ecological risk

##### Sediment quality guidelines

Numerical sediment quality guidelines (SQGs) were applied in the study to assess the ecotoxicological sense of trace element concentrations in sediments (Macdonald et al. 2000, Burton 2002). The assessment of sediment contamination with trace elements was based on threshold effect concentration (TEC) and probable effect concentration (PEC) methods. Trace element concentrations at each station were compared with the consensus-based sediment quality guideline values referred to as TEC and PEC (Table 3). In the study, the mean PEC quotient (PECQ) for seven trace elements was determined. For each sediment sample, the mean PECQ was the average of the ratio of each element concentration to its corresponding PEC. If the mean PECQs were <0.5, sediment samples were predicted to be non-toxic, indicating a low potential toxicity to the benthic fauna. If >0.5, sediment samples were toxic, indicating a high potential risk to the benthic fauna (Macdonald et al. 2000, Niu et al. 2009).

##### Potential ecological risk index

The potential ecological risk index (PERI) is also calculated to assess the harm of trace elements in the studied sediments. The potential ecological risk index for trace elements (TE PERI) was calculated based on the following formula (Håkanson 1980; Guo et al. 2010; Fang et al. 2012; Fu et al. 2013):

$$ \mathrm{PERI}={\displaystyle \sum_i^m{\displaystyle {E}_r^i}}={\displaystyle \sum_i^m{\displaystyle {T}_r^i}}\times {\displaystyle {C}_f^i}={\displaystyle \sum_i^m{\displaystyle {T}_r^i}}\times \frac{{\displaystyle {C}^i}}{{\displaystyle {C}_n^i}} $$

where *E*_*r*_^*i*^ is the potential ecological risk of the trace elements, *T*_*r*_^*i*^ is the toxic-response factor of the trace elements, *C*_*f*_^*i*^ is the contamination factor of the trace elements, *C*^*i*^ represents measured values of the trace elements in the sediments, and *C*_*n*_^*i*^ is the background value of the trace elements in the study area.

The toxic response factor of the trace elements *T*_*r*_^*i*^ depends on the sedimentalogical toxic factor (STF) and bioproduction index (BPI) (Håkanson 1980; Tavakoly Sany et al. 2012; Fiori et al. 2013; Krupadam et al. 2006). STF of individual elements which are determined for Zn = 1; Cu, Pb, Ni = 5; Cr = 2, Cd = 30; Hg = 40 (Håkanson 1980; Guo et al. 2010). BPI is related to the trophic status of the system, which affect the bioavailability of metals. The original model proposed for PERI utilized total nitrogen content and organic matter for sediment bioproduction determination (Håkanson 1980). In the study, BPI was calculated as the nitrogen content in the regression line for the organic matter content value of 10 % (Håkanson 1980; Tavakoly Sany et al. 2012). The value of BPI was equal to 45. *T*_*r*_^*i*^ was calculated for each trace elements as follows (Fiori et al. 2013):

$$ \begin{array}{l}\mathrm{Z}\mathrm{n}=1\times {\left(5/\mathrm{B}\mathrm{P}\mathrm{I}\right)}^{\frac{1}{2}};\mathrm{C}\mathrm{r}=2\times {\left(5/\mathrm{B}\mathrm{P}\mathrm{I}\right)}^{\frac{1}{2}};\mathrm{C}\mathrm{u},\kern0.5em \mathrm{P}\mathrm{b},\kern0.5em \mathrm{N}\mathrm{I}=5\times {\left(5/\mathrm{B}\mathrm{P}\mathrm{I}\right)}^{\frac{1}{2}};\mathrm{C}\mathrm{d}=30\times {\left(5/\mathrm{B}\mathrm{P}\mathrm{I}\right)}^{\frac{1}{2}};\mathrm{H}\mathrm{g}=40\times {\left(5/\mathrm{B}\mathrm{P}\mathrm{I}\right)}^{\frac{1}{2}}\\ {}\end{array} $$

The contamination factor for individual elements was estimated to compare the concentration of elements in sediment with their geochemical background: *C*_*n*_^*i*^ for Zn = 48 mg kg^−1^ d.m.; Cu = 6 mg kg^−1^ d.m.; Ni, Cr = 5 mg kg^−1^ d.m.; Pb = 10 mg kg^−1^ d.m.; Cd = 0.5 mg kg^−1^ d.m.; and Hg = 0.05 mg kg^−1^ d.m. of the bottom sediments (Bojakowska 2001). The contamination degree (*C*_d_) was assessed based on the sum of all contamination factors (Guo et al. 2010; Tavakoly Sany et al. 2012; Manoj and Padhy 2014).

#### Statistical analysis

Statistical analyses including mean, median, standard deviation, minimum, maximum, and variation coefficient (CV%) were determined. The normality of data was checked by the Kolmogorov-Smirnov (K-S) test. Logarithmic transformation was applied to normalize abnormal data. In order to identify the relationships between analyzed parameters in sediments, Pearson correlation coefficient and regression analysis were used. All statistical analyses were preformed with Statistica 12.5 software.

#### Geostatistical analysis

Maps of trace elements and toxicity spatial distribution were created with the Surfer software version 11 based on GPS values obtained from each station. Kriging method was used as a spatial interpretation technique to make distribution maps. This method produces visually appealing maps from irregularly spaced data. Kriging was custom-fitted to a date set by specifying the appropriate return variogram model which was linear model (Delavar and Safari 2016).

## Results

### Physicochemical properties of bottom sediments

The physicochemical parameters of bottom sediments in the Rybnik reservoir are presented in Table 1. At most stations (66 % of samples), sand dominated with low content of organic C (0.56–62.1 g kg^−1^). The silt and clay fractions were dominant in, respectively, 10 and 24 % of the samples. The highest amounts of clay were found at stations situated in the inlet zone and outside the dike. The sediments were characterized by reaction ranging from very acid to slightly alkaline (4.2–7.8). The study showed that the sediments in the inlet zone were characterized by acid or slightly acid reaction, while the sediments in the outlet zone (near the dam) showed neutral or alkaline reaction. Sediments showed high diversity in the basic chemical properties. Organic C, total N, Fe, and Mn occurred in the sediments in a wide range of concentrations (Table 1). Carbon to nitrogen ratios (C/N) in the study was between 10 and 42.

**Table 1:** Basic properties of bottom sediments

Parameters	Grain size (mm) %			pH	C	TN	C:N	Fe	Mn
	Sand	Silt	Clay	KCl	g kg^−1^ d.m		–	g kg^−1^ d.m.	
Minimum	12	0	0	4.2	0.90	0.09	10	1.75	0.04
Maximum	99	46	57	7.8	211.6	10.36	42	43.0	3.02

**Fig. 2:**
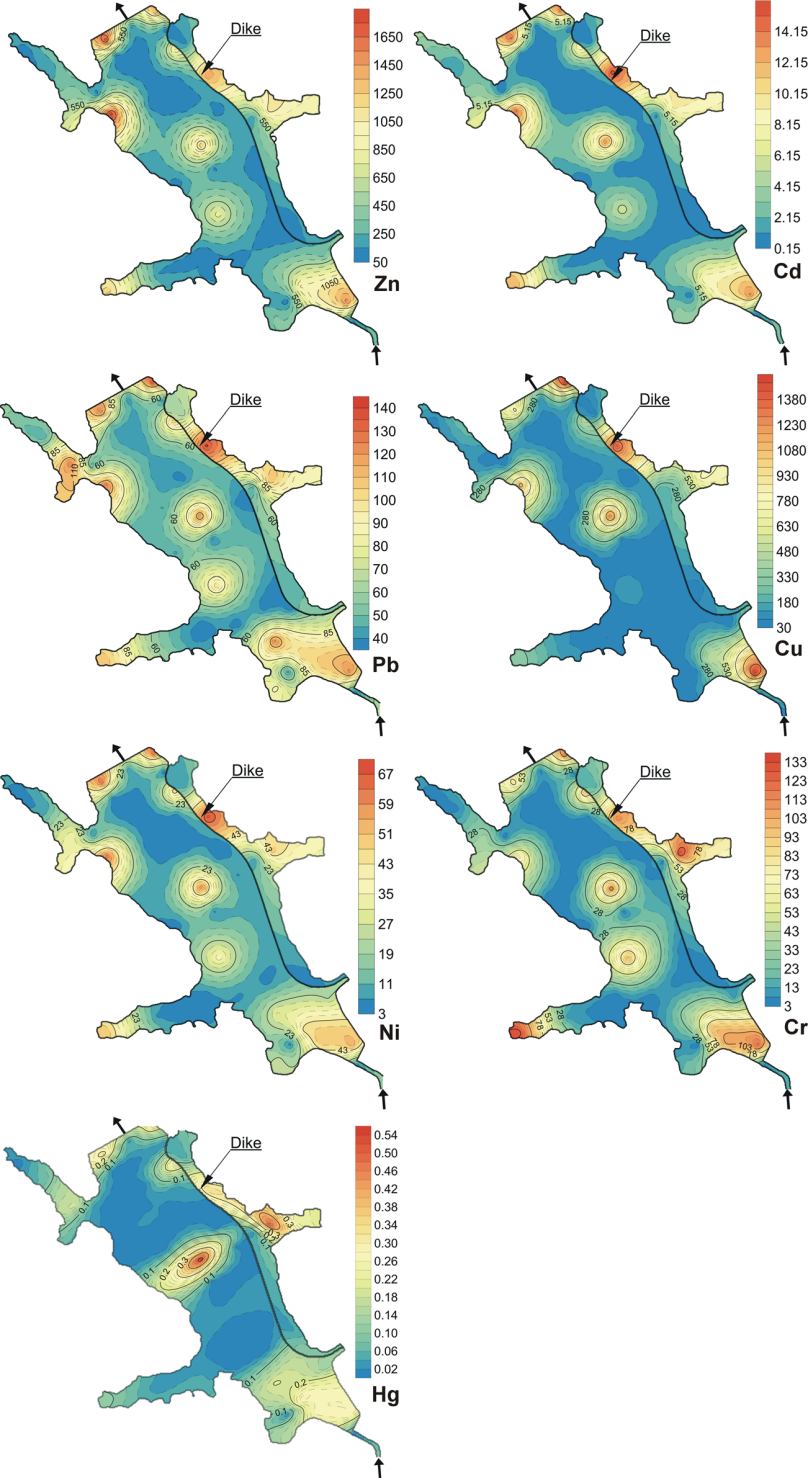
Spatial distribution of trace elements in the sediments

Trace element concentration in the sediments ranged from 79.74 to 1796 mg Zn kg^−1^ d.m., from 0.10 to 15.67 mg Cd kg^−1^ d.m., from 35.69 to 136.8 mg Pb kg^−1^ d.m, from 3.29 to 68.75 mg Ni kg^−1^ d.m., from 33.50 to 1506 mg Cu kg^−1^ d.m., from 2.92 to 132.7 mg Cr kg^−1^ d.m., and from 0.007 to 0.537 mg Hg kg^−1^ d.m. (Table 2). The mean concentration of the elements in the surface layer of the sediments was in the following order: Zn > Cu > Pb > Cr > Ni > Cd > Hg. In the study, significant differences in metal concentrations in the sediments were observed. The computed coefficients of variation (CV) for individual elements were as follows: Cu—157 %; Cd, Cr—132 %; Hg—124 %; Zn—114 %; Ni—102 %; Pb—50 % (Table 2). However, spatial distribution of trace elements in the sediments was similar (Fig. 2). In general, the highest metal concentrations in the sediments were found in the outlet zone located near the dam (stations: R-43, R-45), in the inlet part of the reservoir (stations: R-12, R-13, R-14, R-15), and outside the dike (station: R-47) (Figs. 1 and 2). Higher metal contractions were found also in the middle part of the reservoir (stations: R-27, R-29) (Figs. 1 and 2). The maximum metal concentrations in samples were found in stations: R-38 (Zn), R-47 (Cd, Pb, Ni), R-12 (Cu), R-04 (Cr), and R-29 (Hg). The minimum metal concentrations in the sediments were found in stations: R-39 (Zn, Ni, Cr), R-37, R-19 (Cd, Hg), R-11 (Pb), and R-15 (Cu) (Figs. 1 and 2).

**Table 2:** Concentration of trace elements in bottom sediments

Parameters	Zn	Cd	Pb	Ni	Cu	Cr	Hg	Mean PECQs
	mg kg^−1^ d.m							
Mean	439.4	3.72	67.59	20.43	258.3	32.21	0.106	0.68
Median	152.2	0.98	48.22	7.79	63.26	6.36	0.033	0.26
SD	503.1	4.92	33.79	20.88	406	42.36	0.131	0.82
Minimum	79.74	0.10	35.69	3.29	33.50	2.92	0.007	0.13
Maximum	1796	15.67	136.8	68.75	1506	132.7	0.537	2.78
CV%	114	132	50	102	157	132	124	120

### Assessment of sediment contamination and ecological risk

#### Geochemical quality classes

The assessment of sediment contamination with trace elements was based on Bojakowska’s geochemical quality classes of bottom sediments (2001) (Table 3). According to this criterion, 56 % of the collected sediment samples was classified into class II (moderately contaminated sediments) and 20 % of the samples into class III (contaminated sediments). Sediment samples collected from 12 (24 % of samples) stations R-47, R-45, R-43, R-38, R-36, R-29, R-14, R-12, R-13, R-9, R-8, and R-4 had class IV classification (highly contaminated sediments) (Figs. 1 and 2).

**Table 3:** Qualitative classification of bottom sediments

Guidelines	Zn	Cd	Pb	Ni	Cu	Cr	Hg
Geochemical quality classes^a^ (mg kg^−1^ d.m.)							
Class I	125	0.7	30	16	20	50	0.2
Class II	300	3.5	100	40	100	100	0.7
Class III	1000	6	200	50	300	400	0.7
Class IV	>1000	>6	>200	>50	>300	>400	>0.7
Sediment quality guidelines^b^ (mg kg^−1^ d.m.)							
TEC	121	0.99	35.8	22.7	31.6	43.3	0.18
PEC	459	4.98	128	48.6	149	111	1.06

^a^Bojakowska (2001)

^2^Macdonald et al. (2000)

#### Sediment quality guidelines

The sediment samples were predicted to be non-toxic if the measured concentrations of trace elements were lower than the corresponding TECs (Table 3). Similarly, samples were predicted to be toxic if the measured concentrations of trace elements were higher than the corresponding PECs (Table 3). Samples with metal concentrations between the TEC and PEC were predicted to be neither toxic nor non-toxic (Macdonald et al. 2000). The incidence of toxicity of these samples is greater than that which occurs at the TEC concentration but lower than that which occurs at the PEC concentration (Macdonald et al. 2000). In the case of all metals in the sediments, 30 % (Cu), 26 % (Zn, Cd), 18 % (Ni), 8 % (Cr), and 4 % (Pb) of samples were above the PEC guideline for these metals. TEC values were exceeded in the case of Pb (100 % of samples), Cu (70 % of samples), Zn (46 % of samples), Hg (37 % of samples), Cd (24 % of samples), Cr (18 % of samples), and Ni (14 % of samples).

**Fig. 3:**
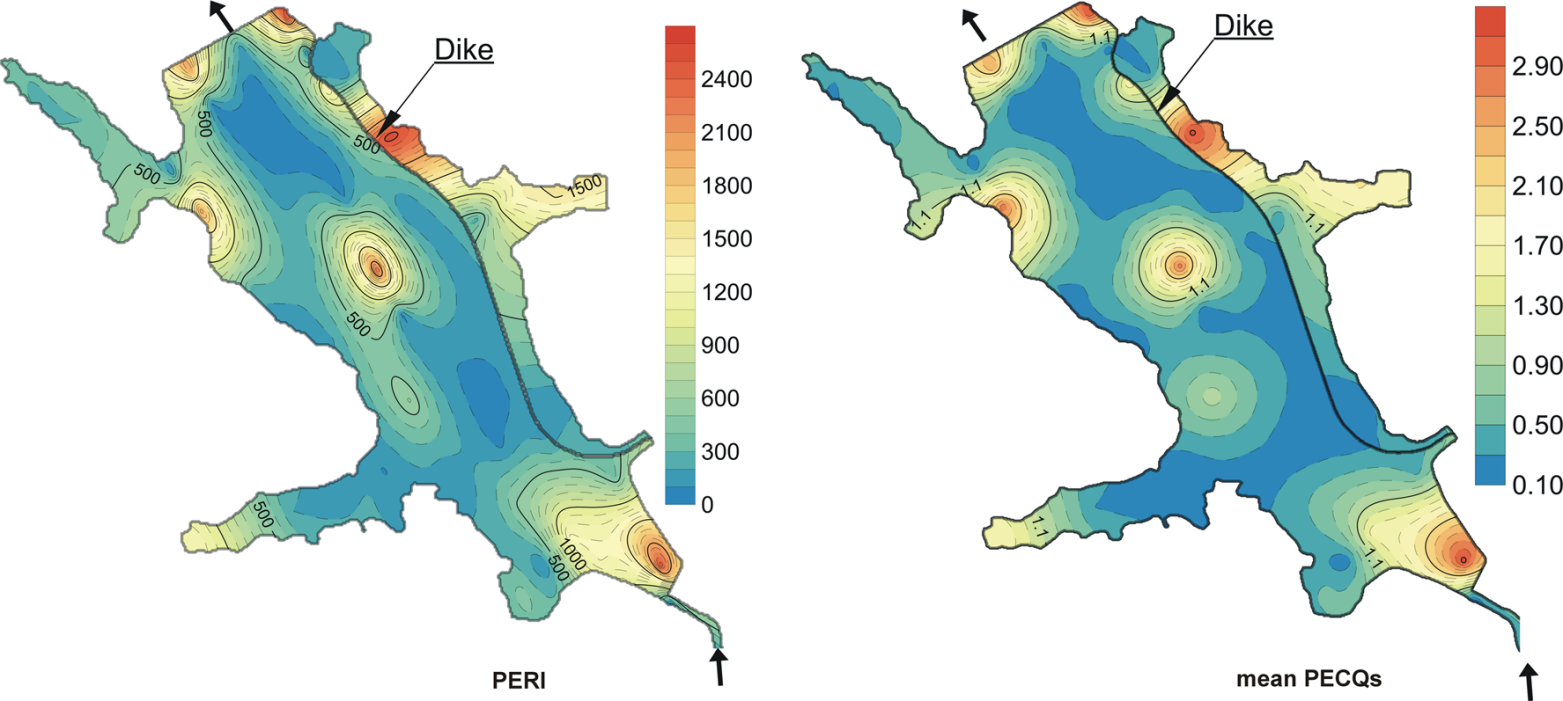
Spatial distribution of mean PECQ values and PERI of seven metals

The mean PECQ provides a basis for assessing the potential effects of sediment-associated contaminants when they occur in a complex mixture (Farkas et al. 2007; Gonçalves et al. 2013; Veses et al. 2013; Fu et al. 2013; Baran and Tarnawski 2015). The mean PECQs of trace elements ranged from 0.13 to 2.78 (Table 2, Fig. 3). The highest mean values of PECQs were found at stations R-12 (inlet part of the reservoir), R-45 (in the outlet zone), and R-47 (outside the dike). The lowest mean values of PECQs were characteristic for stations R-39, R–37, and R-11. It means that 68 % of sediment samples were potentially non-toxic and 32 % of samples were potentially toxic. Ingersoll et al. (2001) used four ranges of the mean PECQs for ranking samples in terms of incidence of toxicity: <0.1, 0.1–<0.5, 0.5–<1.0, and >1.0. These values related to the probability that 10, 17, 56, 97, and 100 % of sediments with mean PECQs, respectively, were toxic in amphipod survival bioassays. In these ranges, 70 % of samples showed mean PECQ values between 0.1 and <0.5, 6 % of samples between 0.5 and <1.0, and 24 % of samples presented mean PECQ values greater than 1. The mean PECQ values obtained in samples R-4, R-8, R-9, R-12, R-13, R-14, R-29, R-38, R-43, R-45, and R-47 were greater than 1, clearly indicating a significant potential risk to benthic fauna. The mean PECQ for individual metal was decreased the following sequence: Cd > Cu > Zn > Pb > Ni > Cr > Hg.

#### Contamination degree and potential ecological risk index

The contamination factor (*C*^*i*^_*f*_) and contamination degree (*C*_d_) were used to assess the anthropogenic contribution of trace elements in the sediments (Tables 4 and 5). The mean values of *C*^*i*^_*f*_ for Cu, Zn, Cd, and Pb were higher than 6 suggesting these trace elements are coming from anthropogenic sources. The contamination factor for Ni and Cr reached considerable mean values indicating that the sediments had considerable anthropogenic inputs of these elements. The mean value of contamination factor for Hg was between 1 ≤ *C*^*i*^_*f*_ < 3 which is indicative of moderate sediment pollution with this element (Tables 4 and 5). Sediments low polluted with Hg and Cr was dominant, they constituted 60 and 48 % of the collected samples. For Ni and Zn results showed that 44 % of sediment samples were moderately polluted. Sediments considerable polluted (44 % of samples) were dominated for Pb. Sediments very high polluted with Cd and Cu were dominant, they constituted 36 and 92 % of the collected samples. The mean values of *C*^*i*^_*f*_ of trace elements formed the following series in descending order: Cu > Zn > Cd > Pb > Cr > Ni > Hg (Table 4). The contamination degree for trace elements in the sediments was within the range from 14.2 to 358 (Table 4). In the study, sediments with moderate, considerable, and very high pollution levels constituted respectively 12, 42, and 46 % of the collected samples.

**Table 4:** Contamination degree and potential ecological risk index of sediments

	Zn	Cd	Pb	Ni	Cu	Cr	Hg	
Parameters	*C* ^*i*^_*f*_^a^							∑ *C* ^*i*^_*f*_ = *C*_*d*_^c^
Mean	9.16	7.44	6.76	4.09	43.1	5.37	2.13	77.9
Median	3.17	1.97	4.82	1.56	10.5	1.06	0.66	26.3
Minimum	1.66	0.21	3.57	0.66	5.58	0.49	0.14	14.2
Maximum	37	31	14	14	251	22	11	358
Parameters	*E* ^*i*^_*r*_^b^							PERI
Mean	3.20	75.2	11.42	6.91	72.8	4.32	28.7	227
Median	1.11	19.9	8.15	2.63	17.8	0.85	8.86	91.6
Minimum	0.58	2.08	6.03	1.11	9.43	0.39	1.83	27.3
Maximum	13.1	316	23.1	23.2	424	18.1	145	950

^a^Contamination factor

^b^Potential of ecological risk index for individual element

^c^Contamination degree

**Table 5:** Classification for contamination degree and PERI

Contamination factor (*C* ^*i*^_*f*_)	Classification	Contamination degree (*C*_*d*_)	Classification
*C* ^*i*^_*f*_ < 1	Low	*C*_d_ < 8	Low
1 ≤ *C* ^*i*^_*f*_ < 3	Moderate	8 ≤ *C*_d_ < 16	Moderate
3 ≤ C^i^_f_ <6	Considerable	16 ≤ *C*_*d*_ < 32	Considerable
C^i^_f_ ≥ 6	Very high	*C*_d_ ≥ 32	Very high
Scope of potential of ecological risk index *E* ^*i*^_*r*_ for individual element	Classification	Scope of potential ecological risk index (PERI)	Classification
*E*_*r*_ < 40	Low	PERI < 150	Low grade
40 ≤ *E*_*r*_ < 80	Moderate	150 ≤ PERI < 300	Moderate
80 ≤ *E*_*r*_ < 160	Higher	300 ≤ PERI < 600	Severe
160 ≤ *E*_*r*_ < 320	Much higher	600 ≤ PERI	Serious
320 ≤ *E*_*r*_	Serious		

The calculated the TE PERI values of the present sediment are summarized in Table 4. The PERI for the trace elements ranged from 27.3 to 950 with a mean of 227 (Table 4). In the studies, PERI of more than 600 indicates a serious potential ecological risk, whereas PERI of less than 150 indicates low potential ecological risk (Table 5) (Guo et al. 2010; Tavakoly Sany et al. 2012, Fang et al. 2012). The results of PERI calculations showed that 70 % of samples had low trace element risks, 6 % of samples were with moderate trace element risks, 12 % of the samples were with severe trace element risks, and 12 % of samples have serious trace element risks. The highest values of PERI above 600 were found at stations R-12 (inlet part of the reservoir), R-45, R-43, R-38 (in the outlet zone), R-47 (outside the dike), and R-29 (middle part of the reservoir). The lowest values of PERI were characteristic for stations: R-37 and R-19. When comparing the potential ecological risk index for individual elements (mean values of *E*_*r*_^*i*^) with the grade classification, the following were found: for Zn Pb, Ni, Cr, and Hg, low potential ecological risk, and for Cd and Cu, moderate potential ecological risk (Tables 4 and 5). The consequence of the mean *E*_*r*_^*i*^ for metals is Cd > Cu > Hg> Pb > Ni > Cr > Zn.

### Sediment toxicity

**Fig. 4:**
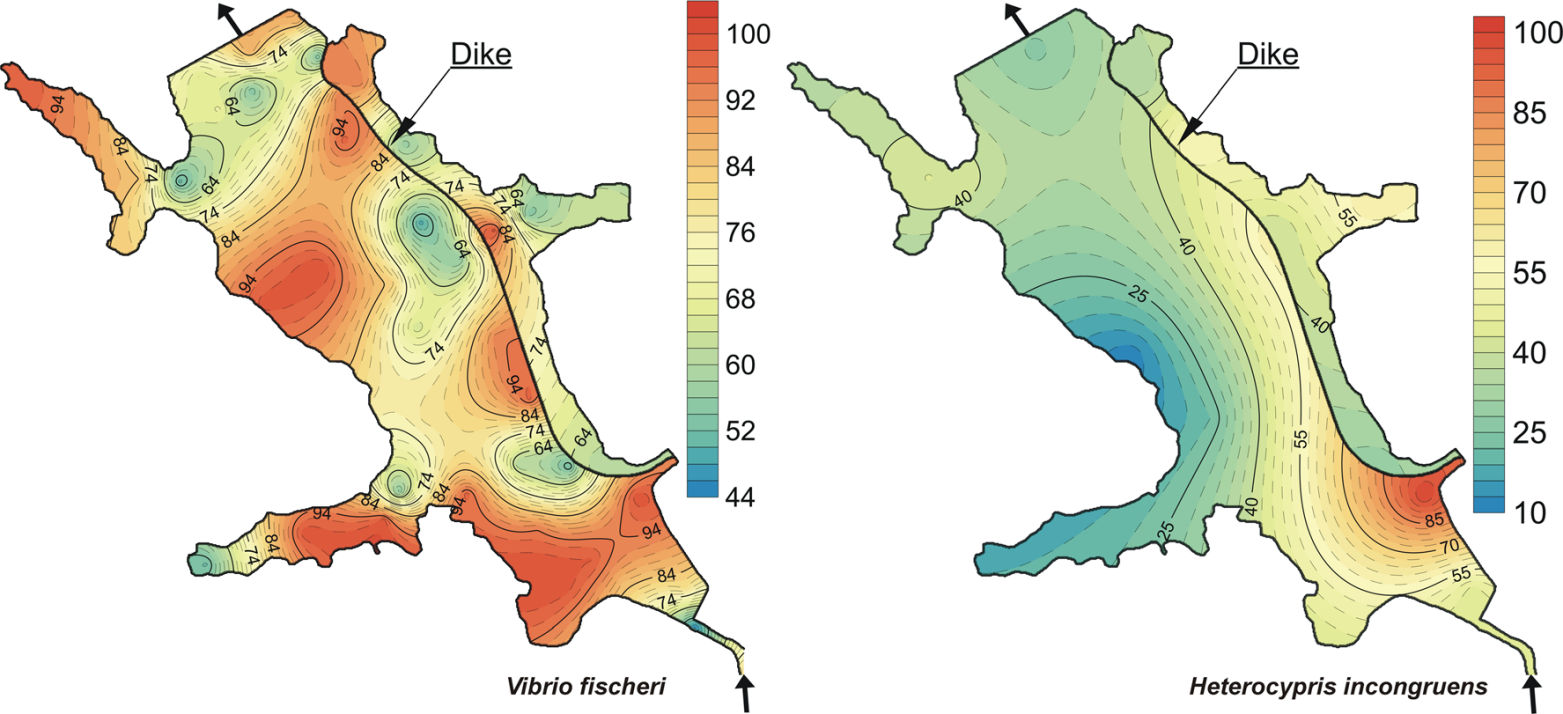
Spatial distribution of luminescence inhibition (%) of *Vibrio fischeri* and growth inhibition (%) of *Heterocypris incongruens* in the sediments

The results of toxicity of the bottom sediments are presented in Table 6 and Fig. 4. In the Microtox test, luminescence inhibition of *V. fischeri* was between 46 and 100 % with a mean percentage effect of 78 % for all the sediment samples. In the Ostracodtoxkit F test, the *H. incongruens* mortality was between 0 and 100 %, whereas growth inhibition was within a range from 10 to 100 % with a mean percentage effect of 14 and 51 %, respectively. The highest toxicity of the bottom sediments in the Microtox test was found at the following stations: R-38 (the outlet zone), R-04 to R-07, R-12 to R-16 (the inlet zone), and R-47 and R-48 (outside the dike) (Fig. 4). Sediments collected at stations R-04, R-05, R-06 (the intel zone), R-25, R-26, R-48 (outside the dike), and R-38, R-41, R-42, and R-43 (the outlet zone) showed the highest toxicity of the bottom sediments in the Ostracodtoxkit test. Sediments collected at stations R-11, R-17, R-18 (*V. fischeri*) and R-17, R-18, and R-19 (*H. incongruens*) showed the lowest toxicity to the test organisms (Fig. 4). Higher toxic responses were recorded in the Microtox test than in the Ostracodtoxkit test. Most sediment samples (96 %) were toxic and highly toxic for *V. fischeri*. For *H. incongruens*, the examined sediments were classified as non-toxic samples (12 %), slightly toxic samples (44 %), toxic samples (38 %), and highly toxic samples (6 %) (Table 6). This result is in agreement with our previous studies on sediments where it was also found that *V. fischeri* showed higher toxic effects than *H. incongruens* (Baran and Tarnawski 2015). In the study by Mankiewicz-Boczek et al. (2008), the highest number of toxic responses was observed for plants in the Phytotoxkit test, then in the Microtox®, and the lowest in the Ostracodtoxkit test. In our other studies, we found a higher number of toxic responses to *V. fischeri* than in the Phytotoxkit test with higher plants (Baran and Tarnawski 2013).

**Table 6:** Toxicity of bottom sediments to *Vibrio fischeri* and *Heterocypris incongruens*

Parameters	*V. fischeri*	*H. incongruens*		Toxicity evaluation	
	Luminescence inhibition	Mortality	Growth inhibition	*V. fischeri*	*H. incongruens*
	Percent effect PE%			% samples	
Mean	78	14	51	Non-toxic^a^—0	Non-toxic—12
Median	82	–	53	Slightly toxic—4	Slightly toxic—44
SD	18	31	26	Toxic—94	Toxic—38
Minimum	46	0	10	Highly toxic—2	Highly toxic—6
Maximum	100	100	100		
CV%	23	–	50		

^a^Non–toxic, PE <20 %; slightly toxic, 20 % ≤ PE < 50 %; toxic, 50 % ≤ PE < 100 %; highly toxic, PE = 100 %

### Correlation coefficient analysis

Correlations between individual trace elements in sediments may be a result of their geochemical connections and can also provide information about sources and pathways of metals (Rinklebe and Shaheen 2014; Shaheen and Rinklebe 2014; Majumder et al. 2015). According to Suresh et al. (2012), high values of the correlation coefficient between the metals suggest that metals have common sources, mutual dependence, and identical behavior during transport. In the studied sediments, all pairs of trace elements were found to be strongly positively correlated (Table 7). Strong positive correlations between individual pairs of trace elements indicate that they may derive from a similar source and move together (Majumder et al. 2015; Baran and Wieczorek 2015).

**Table 7:** Relationships between chemical properties and toxicity of sediments

Parameters	Zn	Cd	Pb	Ni	Cu	Cr	Hg	PECQ	*C*_d_	PERI
Cd	0.99***									
Pb	0.89***	0.90***								
Ni	0.97***	0.97***	0.95***							
Cu	0.88***	0.89***	0.75***	0.84***						
Cr	0.92***	0.93***	0.88***	0.94***	0.77***					
Hg	0.73***	0.76***	0.77***	0.80***	0.68***	0.78***				
Mn	0.93***	0.93***	0.87***	0.92***	0.95***	0.83***	0.70***			
Fe	0.90***	0.91***	0.97**	0.96***	0.73***	0.93***	0.78***			
TOC	0.71***	0.68**	0.76**	0.76**	0.44*	0.76**	0.58**	0.62***	0.50***	0.60***
TN	0.68***	0.66***	0.77***	0.74***	0.44**	0.73**	0.53***	0.59***	0.55***	0.60***
pH	−0.12	−0.14	−0.26	−0.18	0.02	−0.12	−0.12	−0.07	−0.04	−0.08
Sand	−0.80***	−0.81***	−0.92***	−0.88***	−0.60**	−0.86**	−0.73**	−0.76***	−0.71***	−0.76***
Silt	0.71**	0.68**	0.78**	0.75**	0.47***	0.71**	0.50**	0.63***	0.58***	0.62***
Clay	0.79***	0.82***	0.93***	0.90***	0.64***	0.89***	0.73***	0.79***	0.75***	0.80***
*V. f* ^a^	0.55**	0.54**	0.56**	0.56**	0.44**	0.56**	0.43*	0.52**	0.50**	0.52**
*H.i*.^b^	0.36*	0.32*	0.23	0.29*	0.26	0.26	0.03	0.30*	0.28*	0.27

*n* = 50, significant at ***p ≤ 0.001, **p ≤ 0.01, *p ≤ 0.05

^a^ *Vibrio fischeri*

^b^ *Heterocypris incongruens*

The correlation analysis performed on the data enabled the identification of possible common characteristics of trace elements in the sediment, as well as evaluation of the potential of organic matter, pH, and granulometric composition in order to control metal concentration and spatial distribution in the sediments. Total concentrations of trace elements, mean PECQ, *C*_d_, and PERI were significantly positively correlated with organic C, total N, silt, and clay. However, they were significantly negatively correlated with sand (Table 7). Results in Table 7 show that the concentrations of metals in sediments were not significantly correlated with pH. The studies by Baran and Tarnawski (2015) also found that organic matter, sand, and silt significantly affected the distribution of the metals among different geochemical fractions in the sediments of the Rybnik reservoir. The positive relations of trace elements with the organic carbon concentration in the sediments might be attributed to anthropogenic impacts (Farkas et al. 2007; Shaheen and Rinklebe 2014; Baran and Tarnawski 2015). Moreover, the coefficient of correlation between clay and metals was higher than between silt with trace elements, PECQs, *C*_d_, and PERI. This shows that fine particles have the capacity to absorb cations on their surface and raise the level of trace element concentrations (Suresh et al. 2012). In addition, methods of sediment quality assessment using PECQs and PERIs are based on total concentrations of metals, not on their other forms.

An analysis of correlation between the total metal concentration and the results of toxicity to the test organisms was carried out (Table 7). Positive values of the correlation coefficients indicate a relation between metal concentration in the sediments and toxicity to organisms, whereas negative values might suggest that the concentration of a given metal in the sediments did not affect the sample toxicity. A significant positive correlation was found between the total concentration of the metals and luminescence inhibition in *V. fischeri*. Only Zn, Cd, and Ni concentrations in the sediments were significantly positively correlated with the response in *H. incongruens* (Table 7). Research carried out by Płaza et al. (2010) and Baran et al. (2014) also found a significant positive correlation between the concentration of heavy metals in soils polluted with heavy metals and inhibition of *V. fischeri*. Moreover, correlation analyses revealed a significant and positive correlation between the mean PECQs, *C*_d_, and the luminescence inhibition of *V. fischeri* (*r* = 0.52, *r* = 0.50, *p* < 0.01) and growth inhibition of *H. incongruens* (*r* = 0.30, *r* = 0.28, *p* < 0.05) (Table 7). A significant positive correlation was found also between trace element PERIs and luminescence inhibition in *V. fischeri* (*r* = 0.52, *p* < 0.01).

**Fig. 5:**
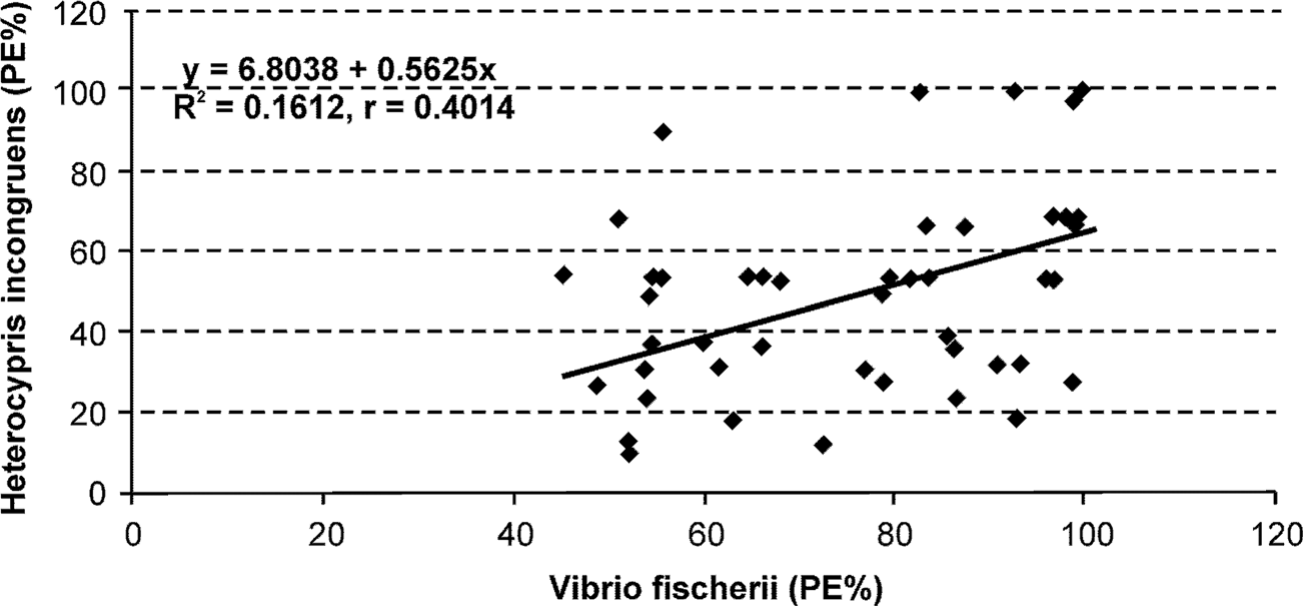
Data pairs correlations of the luminescence inhibition (%) of *Vibrio fischeri* and growth inhibition (%) of *Heterocypris incongruens* in the sediments

Significant correlation was found between luminescence inhibition in *V. fischeri* and growth inhibition in *H. incongruens* (*r* = 0.4014, *p* < 0.01), so it would seem that correlation analysis showed a similar sensitivity to toxicants in the sediments (Fig. 5). However, the outcome of this analysis which is shown in Fig. 5 indicates that correlation between responses of organisms was statistically low significant and explains only 16 % o the variability.

## Discussion

This study used four methods to assess the trace element pollution: SQG (mean PECQ), *C*_d_, PERI, and two bioassays. The mean PECQ was introduced to predict the toxicity of the sediment samples (Cevik et al. 2009; Li 2014). Contamination degree provide over all information about sediment contamination with trace elements (Manoj and Padhy 2014). The PERI method can be used to evaluate the combined pollution risk of an aquatic system through a toxic-response factor for a given substance (Håkanson 1980). Bioassays provided us with information concerning biological impacts of metals in the sediment (actual toxicity of bottom sediment) (Wadhia and Thompson 2007; Fu et al. 2013). Generally, these ecological assessment methods showed both similar and different profiles for the level of trace elements among different sampling sites. Correlation analysis revealed strong significant positive correlations of mean PECQ between PERI (*r* = 0.98, *p* < 0.001), *C*_d_ (*r* = 0.97, *p* < 0.001), and between PERI and *C*_d_ (*r* = 0.98, *p* < 0.001) (Table 7) suggesting that the three methods were highly compatible for evaluation of the polluted sites. Moreover, taking above methods into account, it was shown that Cd and Cu are responsible to the greatest degree for the ecological risk of sediments. These correlations indicate that in the studied reservoir the responsible substances for PERI (TOC, TN) and considered trace elements may have the same source. The main sources of water contamination in the reservoir and the Ruda and Nacyna rivers (main rivers which feed the reservoir) include the following: treated industrial sewage emitted by the Rybnik power plant, municipal sewage, rain wastewaters, sewage from the water treatment plant, cooling tower blowdowns, and dry precipitation (Loska and Wiechuła 2003; Baran and Tarnawski 2015; Baran et al. 2015). The study showed that increases in lead concentrations in sediments were connected with the inflow of contaminated water of the Ruda river and long-range transport (Wiechuła et al. 2005; Koniarz et al. 2014). The Ruda river is a receiver of municipal sewage coming from municipal treatment plants and other industrial plants. In the study of Kostecki (2004), Kostecki and Kowalski (2004), similarly as in our study, found strong differentiation spatial distribution of metals, organic matter, and mineral particles in the sediments of Rybnik reservoir. This effect is caused by hydrodynamic conditions which are a result of anthropopression, as the supplementary water mass movement. When the mixion of the water mass as a results of discharge water from power plant (thermal pollutant) is stronger than natural water movement (Ruda river), in the Rybnik reservoir, disruption of natural limnological factors and the formation of zones with high concentration of pollutants in the sediments (Kostecki 2004) were observed. The changes in water temperature result in vertical circulation of water masses and cause variable conditions at the site where the reservoir water mixes with inflowing river water. Anthropogenically forced circulation of water in the reservoir contributes also to the occurrence of turbulence and horizontal reverse current-variable motion of bottom and surface layers.

The correlation analysis also showed a positive relation between the m-PECQ, *C*_d_, PERI values, trace elements, and the response of *V. fischeri* and *H. incongruens*. However, these relationships were weak (for *H. incongruens*), moderate (*for V. fischeri*), or insignificant (for *H. incongruens*) (Table 7). This indicated that PERI, *C*_d_, m-PECQ, and bioassays gave slightly different results due to different assessing mechanisms. Firstly, the m-PECQ method is based on sediment quality guidelines (PEC values) while PERI takes into account different background values of geography, but both methods are connected with biological response. Bioassays indicate general toxicity of the samples as a result of complexity of the samples, but do not identify the cause of the observed toxic responses (Wadhia and Thompson 2007; Chapman et al. 2002; Tuikka et al. 2011; Fu et al. 2013). Therefore, weak or insignificant correlations between metal concentration in the sediments and response of organisms suggested that there are other factors that contribute to the toxicity of the sediments, such as organic compounds, e.g., PAHs, PCBs, PCDDs, and PCDFs (Jancewicz et al. 2012; Urbaniak et al. 2010, 2013), and inorganic compounds, e.g., arsenic (Widziewicz and Loska 2012). Additionally, interactions between pollutants may result in antagonistic or synergistic toxic effects that are difficult to predict (Narracci et al. 2009; Fu et al. 2013; Witt et al. 2014). The PERI application in the study also may explain the low value of the correlation coefficients between the metals and the response of test organisms. This study found the high value of bioproduction index (BPI = 45), which indicated that the reservoir environment would be classified as hypertrophic (Fiori et al. 2013). The value of the trophic state index (TSI) classified Rybnik reservoir as a eutrophic or hipereutrophic basin (Kowalski and Mazierski 2008). In a eutrophic ecosystem, trace elements are less bioavailable due to complexation effects and to biological dilution (Hakanson 2003). Another author showed that the eutrophication Guanabara bay was able to reduce the PERI for trace elements, despite the high concentrations found in sediments (Fiori et al. 2013). Many authors have shown that organisms may have different sensitivities to various substances (Latif and Licek 2004; Mankiewicz-Boczek et al. 2008; Tuikka et al. 2011; Gonçalves et al. 2013; Baran and Tarnawski 2013, 2015). The main route of substances to bacteria is through diffusion of free dissolved chemicals from sediments. Another potential route is through sorption of lipophilic compounds to humic acids (Tuikka et al. 2011). The main exposure route for *H. incongruens* is the contact with chemicals in the sediments and in the overlying water, and through feeding on microalgae (Chapman et al. 2002; Chial and Persoone 2002; Ruiz et al. 2013). In this study, while estimating the sensitivity of the performed bioassays, a higher number of toxic responses and significant correlations were recorded for *V. fischeri* than for *H. incongruens* (Fig. 4, Tables 6 and 7). Thirdly, the test organisms were exposed to substances dissolved in water (Microtox) as well as to substances absorbed on the surface of solid particles (Ostracodtoxkit). The regression line for the two organisms confirms the overall different sensitivities of the *V. fischeri* and *H. incongruens* for to toxicants in the sediments (Fig. 5). In our observation, differences between responses of the organisms were due to sorption of elements by the inorganic and organic particles, and their lower bioavailability for the *H. incongruens* (Leitgib et al., 2007; Płaza et al. 2010). Moreover, our study showed that bottom sediments from the Rybnik reservoir had a high concentration of organic matter, which influenced the solubility and mobility of the trace elements in the organisms (Baran and Tarnawski 2015). We think that higher toxicity of sediment samples for *V. fischeri* than for *H. incongruens* was also connected with the procedure of 81.9 % screening test (Microtox). Solid phase of sediment in contact with distilled water (shaking mechanically for 24 h) can cause water extracts with higher concentrations of potentially toxic substances.

On the other hand, the tests using *H. incongruens* correlated with Cd, Ni, and Zn concentrations, m-PECQ, and *C*_d_ may be suggesting that even when elements are to sorption by clay, silt, or C organic, these animals are exposed to them, both for direct contact or for ingestion, which is different from Microtox test, where only the dissolved elements are bioavailable by bacteria. Chial and Persoone (2002) used the *H. incongruens* indicating that the ostracod mortality in Zn-polluted soils was a result of the non-soluble toxicants bound to the solid-phase particles, rather than of those that had dissolved in the water phase. Baran and Tarnawski (2015) and Merchan et al. (2014) showed also a higher toxicity in solid phases than in liquid phase of sediment. Given the information obtained above, it is now possible to suggest that in the ecological risk assessment, biotests on the solid phase are necessary to complement biotests on leachates. Biotests performed on leachates or extracts to assess only the impact of soluble (bioavailable forms) compounds. The used of two biotests allowed one to assess the potential of toxic contaminants, considering several exposure routes (solid and liquid phase) and different endpoint effects based on sensitivity (acute and chronic toxicity).

## Conclusion

Spatial distributions of trace elements in the sediments were the key step to understanding the contamination in the reservoir system. Despite the high diversity in metal concentration in the sediments, the spatial distribution of trace elements in the sediments was very similar. Moreover, the strong positive correlations between individual pairs of trace elements indicate that they may derive from a similar source and move together. The most polluted sediments are found at points in the inlet zones, near the dam (outlet zones) and outside the dike—point source of metals. The contamination of sediments by metals is probably connected with the discharge of cooling water from the power plant and with an inflow of contaminated water of the Ruda River and long-range transport. The specific hydrodynamic condition in the reservoir could explain the spatial distribution of trace elements and toxicity in the sediments. According to mean probable effect concentration quotients (m-PECQs), 68 % of the samples were potentially non-toxic and 32 % of the samples were potentially toxic. Potential ecological risk index (PERI) values suggested that 70 % of the sediment sampling sites exhibited low ecological risk from metal pollution while 24 % of the samples had severe and serious risk. Based on our combined evaluation, we believe that Cd and Cu in the sediment samples were the main cause of the adverse biological effects. Sediments of the Rybnik reservoir are toxic, but the two test organisms showed a different sensitivity. Higher toxic responses were observed in many samples with the bacterial test than with the ostracod crustacean assay. For *V. fischeri*, 96 % of the sediment samples had effect percentages >50 % whereas for *H. incongruens*, 12 % of the sediments were not toxic and 44 % had effect percentages >50 %.

To sum up, bioassays used in this study with different organisms, toxicity endpoints, and types and time of exposure proved more efficient in assessing the sediment mixture-toxicity than any of the tests alone. Application of mean probable effect concentration quotients and potential ecological risk index based on measures of trace elements improved the correct classification of samples into toxic or non-toxic. In order to perform a complex assessment of the environmental impact of metal pollution, both chemical and ecotoxicological analysis should be carried out. A similar conclusion has been drawn from several other sediment studies (Davoren et al. 2005; Mankiewicz-Boczek et al. 2008; Mamindy-Pajany et al. 2011; Tuikka et al. 2011; Fu et al. 2013; Baran et al. 2015; Baran and Tarnawski 2013, 2015). Furthermore, this integrative approach allowed a comprehensive evaluation of the quality of sediment and in the future will allow the implementation of remediation strategies to obtain a good ecological potential of reservoir. In addition combination of sediment assessment can be constructive in designing and planning strategic sediment management programs and policies (Tavakoly Sany et al. 2012; Manoj and Padhy 2014). However, more studies are needed to investigate the toxicity of multi-element pollution (organic pollutants, forms of pollutants, different biotests, water properties) in sediments to advance knowledge environmental risk assessment and reservoir remediation.

## References

[CR1] Ayas Z, Ekmekci G, Yerli SV, Ozmen M (2007). Heavy metal accumulation in water, sediments and fishes of Nallihan Bird Paradise, Turkey. J Environ Biol.

[CR2] Baran A, Tarnawski M (2013). Phytotoxkit/Phytotestkit and Microtox® as tools for toxicity assessment of sediments. Ecotox Environ Safe.

[CR3] Baran A, Tarnawski M (2015). Assessment of heavy metals mobility and toxicity in contaminated sediments by sequential extraction and a battery of bioassays. Ecotoxicology.

[CR4] Baran A, Wieczorek J (2015). Application of geochemical and ecotoxicity indices for assessment of heavy metals content in soils. Arch Environ Prot.

[CR5] Baran A, Czech T, Wieczorek J (2014) Chemical properties and toxicity of soils contaminated by mining activity. Ecotoxicology 2(7):1234–1244 10.1007/s10646-014-1266-y PMC4131150 24903806

[CR6] Baran A, Tarnawski M, Michalec B (2015) Assessment of metal leachability and toxicity from sediment potentially stored on land. Water SA 41(5).doi: 10.4314/wsa.v41i5.03

[CR7] Bastami KD, Neyestami M, Shemirani F, Soltani F, Haghparast S, Akbari A (2015). Heavy metal pollution assessment in relation to sediment properties in the coastal sediments of the southern Caspian Sea. Mar Pollut Bull.

[CR8] Besser JM, Brumbaugh WG, Ingersoll ChG (2014) Characterizing toxicity of metal-contaminated sediments from mining areas. Appl Geochem doi:10.1016/j.apgeochem.2014.05.021 10.1002/etc.4276 30239039

[CR9] Bojakowska I (2001). Criteria for evaluation of water sediments pollution. Polish Geological Review.

[CR10] Burton A (2002). Sediment quality criteria in use around the world. Limnology.

[CR11] Cevik F, Goksu MZL, Dereci OB, Findik O (2009). An assessment of metal pollution in surface sediments of Seyhan Dam by using enrichment factor, geoaccumulation index and statistical analyses. Environ Monit Assess.

[CR12] Chapman PM, Ho KT, Munns WR, Soloman K, Weinstein MP (2002). Issues in sediment toxicity and ecological risk assessment. Mar Pollut Bull.

[CR13] Chial BZ, Persoone G (2002). Cyst-based toxicity tests XIV. Application of the ostracod solid phase microbiotest for toxicity monitoring of river sediments in Flanders (Belgium). Environ Toxicol.

[CR14] Davoren M, Ní Shúilleabháin S, O’ Halloran J, Sheehan D, O’Brien NM, van Pelt F, Mothersill C (2005). A test battery approach for the ecotoxicological evaluation of estuarine sediments. Ecotoxicology.

[CR15] Delavar MA, Safari Y (2016). Spatial distribution of heavy metals in soils and plants in Zinc Town, northwest Iran. Int J Environ Sci Technol.

[CR16] Du Laing G, Rinklebe J, Vandecasteele B, Meers E, Tack FMG (2009). Trace metal behaviour in estuarine and riverine floodplain soils and sediments: a review. Sci Total Environ.

[CR17] Fang SB, Jia XB, Yang XY, Li YD, An SQ (2012). A method of identifying priority spatial patterns for management of potential ecological risks posed by heavy metals. J Hazard Mater.

[CR18] Farkas A, Erratico C, Vigano L (2007). Assessment of the environmental significance of heavy metal pollution in surficial sediments of the River Po. Chemosphere.

[CR19] Fiori C, Rodrigues A, Santeli R, Corderio R, Carvalheira R, Araujo P, Castilhos Z, Bidone E (2013). Ecological risk index for aquatic pollution control: a case study of coastal water bodies from the Rio de Janerio State, southeastern Brazil. Geochem Brasiliensis.

[CR20] Förstner U, Salomons W (2010). Sediment research, management and police. J Soil Sediment.

[CR21] Fu J, Hu X, Tao X, Yu H (2013). Risk and toxicity assessments of heavy metals in sediments and fishes from the Yangtze River and Taihu Lake, China. Chemosphere.

[CR22] Gao H, Bai J, Xiao R, Liu P, Jiang W, Wang J (2013). Levels, source and risk assessment of trace elements in wetland soils of a typical shallow freshwater lake, China. Stoch Env Res Risk A.

[CR23] Gonçalves SF, Calado R, Gomes NCM, Soares AMVM, Loureiro S (2013). An ecotoxicological analysis of sediment quality in European Atlantic harbor emphasizes the current limitation of the Water Framework Directive. Mar Pollut Bull.

[CR24] Guo W, Liu X, Liu Z, Li G (2010). Pollution and potential ecological risk evaluation of heavy metals in the sediments around Dongjiang Harbor, Tianjin. Procedia Environ Sci.

[CR25] Håkanson L (1980). An ecological risk index for aquatic pollution control. A sedimentological approach. Water Res.

[CR26] Hakanson L (2003) A general management model to optimize lake liming operations. Lakes & Reservoirs: Research & Management 8(2):105–140

[CR27] Hou D, He J, Lü C, Ren L, Fan Q, Wang J, Xie Z (2013). Distribution characteristics and potential ecological risk assessment of heavy metals (Cu, Pb, Zn, Cd) in water and sediments form Lake Dalinouer, China. Ecotox Environ Safe.

[CR28] Ingersoll CG (2001). Predictions of sediment toxicity using consensus-based freshwater sediment quality guidelines. Arch Environ Contam Toxicol.

[CR29] ISO 14371:2012 Water quality. Determination of fresh water sediment toxicity to Heterocypris incongruens (Crustacea, Ostracoda).

[CR30] Jancewicz A, Dmitruk U, Sosnicki L, Tomczuk U, Bartczak A (2012). Influence of land development in the drainage area on bottom sediment quality in some dam reservoirs. Environmental Pollution Control.

[CR31] Kemble NE (2012). Contaminants in stream sediments form seven Unites States Metropolitan areas. Part II—sediment toxicity to the amphipod *Hyalella azteca* and midge *Chironomus dilutus*. Arch Environ Contam Toxicol.

[CR32] Koniarz T, Tarnawski M, Baran A (2014). Content of lead in bottom sediments of the water reservoir located in urban areas. Logistyka.

[CR33] Kostecki M (2004). Anthropopression impact on the formation of thermal structure on the Rybnik dam-reservoir. Arch Environ Prot.

[CR34] Kostecki M, Kowalski E (2004). Distribution of heavy metals in the Rybnik dam-reservoir bottom sediments. Arch Environ Prot.

[CR35] Kowalski E, Mazierski J (2008). Effects of cooling water discharges from a power plant on reservoir water quality. Ocenol Hydrobiol St.

[CR36] Krupadam RJ, Smita P, Wate SR (2006). Geochemical fraction of heavy metals in sediments of Tapi estuary. Geochem J.

[CR37] Latif M, Licek E (2004). Toxicity assessment of wastewaters, river water and sediments in Austria using cost-effective microbiotests. Environ Toxicol.

[CR38] Leitgib L, Kalman J, Gruiz K (2007). Comparison of bioassays by testing whole soil and their water extract from contaminated sites. Chemosphere.

[CR39] Li J (2014). Risk assessment of heavy metals in surface sediments form the Yanghe River, China. Int J Environ Rs Public Health.

[CR40] Loska K, Wiechuła D (2003). Application of principal component analysis for the estimation of source of heavy metal contamination in surface sediments from the Rybnik reservoir. Chemosphere.

[CR41] Macdonald DD, Ingersoll CG, Berger TA (2000). Development and evaluation of consensus-based sediment quality guidelines for freshwater ecosystems. Arch Environ Contam Toxicol.

[CR42] Majumder RK, Faisal BMR, Zaman MN, Uddin MJ, Sultana N (2015). Assessment of heavy metals pollution in bottom sediment of the Burganga River, Dahaka, Bangladesh by multivariate statistical analysis. Int Res J Environment Sci.

[CR43] Mamindy-Pajany Y, Hamer B, Roméo M, Géret F, Galgani F, Durmisi E, Hurel C, Marmier N (2011). The toxicity of composted sediments from Mediterranean ports evaluated by several bioassays. Chemosphere.

[CR44] Mankiewicz-Boczek J, Nałecz-Jawecki G, Drobniewska A, Kaza M, Sumorok B, Izydorczyk K, Zalewski M, Sawicki J (2008). Application of microbiotest battery for complete toxicity assessment of rivers. Ecotoxicol Environ Saf.

[CR45] Manoj K, Padhy PK (2014). Distribution, enrichment and ecological risk assessment of six elements in bed sediments of a tropical river, Chottanagpur Plateau: a spatial and temporal appraisal. J Environ Prot.

[CR46] Merchan CG, Perrodin Y, Barraud S, Sébastian C, Becouze-Lareure C, Bazin C, Kouyi GL (2014). Spatial variability of sediment ecotoxicity in a large storm water detention basin. Environ Sci Pollut Res.

[CR47] MicrobicsCorporation (1992) Microtox manual toxicity testing handbook. Carlsbad, CA, USA.

[CR48] Narracci M, Cavallo RA, Acquaviva ML, Prato E, Biandolino F (2009). A test battery approach for ecotoxicological characterization of Mar Piccolo sediment in Taranto (Ionian Sea, southern Italy). Environ Monit Assess.

[CR49] Niu H, Deng W, Wu Q, Chen X (2009). Potential toxic risk of heavy metals from sediment of the Pearl River in South China. J Environ Sci.

[CR50] Ostracodtoxkit F (2001). Direct contact toxicity test for freshwater sediments. Standard operational procedure.

[CR51] Persoone G, Marsalek B, Blinova I, Törökne A, Zarina D, Manusadzianas L, Nałecz-Jawecki G, Tofan L, Stepanova N, Tothova L, Kolar B (2003). A practical and user-friendly toxicity classification system with microbiotests for natural waters and wastewaters. Environ Toxicol.

[CR52] Płaza G, Nałęcz-Jawecki G, Pinyakong O, Illmer P (2010). Ecotoxicological and microbiological characterization of soils from heavy metal and hydrocarbon contaminated sites. Environ Monit Assess.

[CR53] Rinklebe J, Shaheen SM (2014). Assessing the mobilization of cadmium, lead, and nickel using a seven-step sequential extraction technique in contaminated floodplain soil profiles along the Central Elbe river, Germany. Water, Air, Soil Pollut.

[CR54] Ruiz F, Abad M, Bodergat AM, Carbonel P, Rodrıguez-Lazaro J, Gonzalez-Regalado ML, Toscan A, Garcı’a EX, Prenda J (2013) Freshwater ostracods as environmental tracers. Int J Environ Sci Technol 10:1115–1128

[CR55] Sayed S, Moussa E, El-Sabagh M (2015). Evaluation of heavy metal content in Qaroun Lake, El-Fayoum, Egypt. Part I: bottom sediments. JRRAS.

[CR56] Shaari H, Azmi S, Sultan K, Bidai J, Mohamag Y (2015) Spatial distribution of selected heavy metals in surface sediments of EAZ of the East Coast of Peninsular Malaysia. Int J Oceanogr doi.10.1155/2015/618074

[CR57] Shaheen SM, Rinklebe J (2014). Geochemical fractions of chromium, copper, and zinc and their vertical distribution in floodplain soil profiles along the Central Elbe. Geoderma.

[CR58] Suresh G, Sutharsan P, Ramasamy V, Venkatachalapathy R (2012). Assessment of spatial distribution and potential ecological risk of the heavy metals in relation to granulometric contents of Veeranam lake sediments India. Ecotoxicol Environ Saf.

[CR59] Tavakoly Sany SB, Salleh A, Sulaiman AH, Sasekumar A, Tehrani G, Rezayi M (2012). Distribution characteristics and ecological risk of heavy metals in surface sediments of west port, Malaysia. EPE.

[CR60] Tuikka AI, Shmitt C, Hoss S, Bandow N, Carsten von der Ohe P (2011) Toxicity assessment of sediments from three European river basins using a sediment contact test battery. Ecotoxicol Environ Saf 74:123–131 10.1016/j.ecoenv.2010.08.038 20833427

[CR61] Urbaniak et al. 2008 PCBs and heavy metals contamination in bottom sediments from three reservoirs of different catchment characteristics. Polish J Environ Stud 17(6): 941-949.

[CR62] Urbaniak M, Zieliński M, Ligocka D, Zalewski M (2010). The comparative analysis of selected persistent organic pollutants (POPs) in reservoirs of different types of anthropopression—Polish and Ethiopian studies. Fresen Environ Bull.

[CR63] Urbaniak M, Zieliński M, Kaczkowski Z, Zalewski M (2013). Spatial distribution of PCDDs, PCDFs and dl-PCBs along the cascade of urban reservoirs. Hydrol Res.

[CR64] Veses O, Mosteo R, Ormad MP, Ovelleiro JL (2013). Sediment quality assessment of two industrialized areas of Spain. Int J Environ Res.

[CR65] Wadhia K, Thompson KC (2007). Low-cost ecotoxicity testing of environmental samples using microbiotests for potential implementation of Water Framework Directive. Trends Anal Chem.

[CR66] Wang C, Liu S, Zhao Q, Dong S (2012). Spatial variation and contamination assessment of heavy metals in sediments in the Manwan Reservoir, Lancang River. Ecotoxicol Environ Saf.

[CR67] Widziewicz K, Loska K (2012). Multivariate statistical analyses on arsenic occurrence in Rybnik reservoir. Arch Environ Prot.

[CR68] Wiechuła I, Loska K, Korus I (2005). Lead partitioning in the bottom sediment of Rybnik reservoir (Southern Poland). Water Air and Soil Poll.

[CR69] Witt A, Kobusińska M, Maciak J, Niemrycz E (2014). Geostatistical methods for estimation of toxicity of marine bottom sediments based on the Gdańska Basin area. Oceanol Hydrobiol St.

